# A Systematic Review of Shoulder Arthroplasty in Parkinson's Disease

**DOI:** 10.1177/24715492231162302

**Published:** 2023-03-13

**Authors:** Patrick J Carroll, Ujash Sheth, Patrick Henry

**Affiliations:** 1 Sunnybrook Orthopaedic Upper Limb, Sunnybrook Health Sciences Centre, Division of Orthopaedic Surgery, 7938 University of Toronto, Toronto, Ontario, Canada

**Keywords:** Shoulder arthroplasty, Parkinson's disease, shoulder replacement

## Abstract

**Background:**

Parkinson's disease is a degenerative neurological disorder that can cause both motor and nonmotor symptoms. Motor symptoms are associated with increasing the patient's falls risk. Shoulder arthroplasty surgery in this patient cohort is associated with more complications than non-Parkinsonian patients. We sought to identify any increase in complications associated with this patient cohort and any surgical considerations that ought to be taken in light of their disease process.

**Methods:**

We performed a systematic review of articles using PubMed, MEDLINE, Cochrane Central, and Google Scholar. All studies which included any shoulder arthroplasty surgery for patients with Parkinson's disease were included.

**Results:**

Complication rates were higher in patients with Parkinson's disease than in the normal arthroplasty cohort in all studies. There was significant heterogeneity between all 8 studies included in the systematic review. Complication rates ranged from 26% to 100%. Complications included subluxation, loosening, malunion, nonunion, scapular notching, stiffness, fracture, baseplate failure, dislocation, and infection. Reoperation rates ranged from 5% to 29%.

**Conclusion:**

Compared to patients without Parkinson's disease undergoing shoulder arthroplasty, patients with Parkinson's disease achieved similar reductions in pain but inferior clinical function. The range of movement was less predictable, and complication rates were significantly higher in Parkinson's disease patients. This study will aid the surgeon and patient regarding surgical intervention, informed consent, and allow the surgeon to anticipate potential complications of shoulder arthroplasty in this patient cohort.

## Introduction

The first articular metal prosthesis ever implanted was likely a shoulder prosthesis.^1,2^ Multiple different shoulder arthroplasties have been performed since then with the Neer anatomical total shoulder arthroplasty (aTSA) and hemiarthroplasty (HA) designs as well as the reverse total shoulder arthroplasty (rTSA).^2–4^ In the United States, shoulder arthroplasty surgery increased by 24% between 2011 and 2014. The proportion of rTSA surpassed aTSA for the first time in 2014, while the proportion of HA procedures declined.^5^ The National Joint Registry of England, Wales, Northern Ireland, and the Isle of Man (NJR) started collecting registry data on shoulder arthroplasty in 2012.^6^ A review of joint registry data has shown that the cumulative revision rate for shoulder arthroplasty is decreasing over time.^7^ Shoulder arthroplasty surgery has shown excellent results with significant improvements in clinical outcome measures.^8^

Parkinson's disease is a common and complex neurological disorder.^9^ It is a neurodegenerative disease with early prominent death of dopaminergic neurons in the substantia nigra pars compacta. The resultant dopamine deficiency within the basal ganglia leads to a movement disorder characterized by classical parkinsonian motor symptoms such as bradykinesia, muscular rigidity, resting tremor, and postural instability. It is also associated with nonmotor symptoms such as constipation, depression, urinary symptoms, orthostatic hypotension, dementia, freezing gait, psychosis, and falls.^9,10^ Parkinson's disease is recognized as the most common neurodegenerative disorder after Alzheimer's disease. The incidence of Parkinson's disease ranges from 10 to 18 per 100 000 person-years.^11^

Patient's with Parkinson's disease undergoing trauma and orthopedic surgery procedures have had increased complications compared to non-Parkinsonian patients. Some authors have recommended arthroplasty over femoral neck fixation for fractures of the femoral neck.^12,13^ Other authors have noted an increased dislocation risk in similar groups of patients and recommend internal fixation.^14,15^ A recent study on patient's undergoing knee arthroplasty showed patient's with Parkinson's disease had an increased risk of complications.^16^

Up until the 21st century, very little was known about shoulder arthroplasty in patients with Parkinson's disease. Since then a number of studies have been performed on the outcome of shoulder arthroplasty in Parkinson's disease. We reviewed the published literature on this topic to aid the surgeon and patient regarding surgical intervention, informed consent, and allow the surgeon to anticipate potential complications of shoulder arthroplasty in patients with Parkinson's disease.

## Methods

This systematic review was conducted according to the methods of the Cochrane Handbook for Systematic Reviews and is reported according to the Preferred Reporting Items for Systematic Reviews and Meta-Analyses (PRISMA) guidelines.^17,18^

### Search Strategy and Eligibility Criteria

The search was performed using PubMed, MEDLINE, Cochrane Central, and Google Scholar. These databases were screened from their inception until August 28, 2020. The Boolean search terms were: “Total shoulder arthroplasty” or “Total shoulder replacement” or “Shoulder arthroplasty” or “Shoulder replacement” or “Reverse shoulder arthroplasty” or “Reverse shoulder replacement” or “Reverse total shoulder arthroplasty” or “Reverse total shoulder replacement” or “Anatomic total shoulder arthroplasty” or “Anatomic total shoulder replacement” or “Anatomic shoulder arthroplasty” or “Anatomic shoulder replacement” or “Shoulder hemiarthroplasty” AND “Parkinson's disease”.

### Study Selection

Two individuals (PC and PH) independently conducted a computerized search of the electronic databases PubMed, EMBASE, Google Scholar, and Cochrane Central. There was no disagreement between the search, and a third reviewer was not necessary.

### Assessment of Eligibility

We included all studies in which patient's with Parkinson's disease received a shoulder arthroplasty procedure for any cause. We did not exclude any studies. Where available patient demographics, complications, implant survivorship, and patient-reported outcome measures were included in quantitative analysis.

### Data Extraction Method

Data were extracted by authors, publication year, study level, study type, demographic data (age, sex, and sample size), type of arthroplasty, follow-up time, outcomes, and range of movement.

### Assessment of Quality

Quality assessment was performed using the Methodological Index for Non-Randomized Studies (MINORS), a validated scoring system for the methodologic quality of comparative and noncomparative nonrandomized surgical studies.^19^ The scoring categories are assigned a rating from 0 to 2, where the ideal score for a comparative study is 24. Categorization of the quality of the MINORS score was based on previous systematic reviews that separated the MINORS into very low: 0 < MINORS score < 6; low: 6 MINORS score < 10; and fair: 10 MINORS score > 14.^20,21^

## Results

A total of 8 studies were identified as being eligible for the study.^22–29^ Patient demographics, clinical outcomes, and complications are outlined in Tables 1 to 3. The studies were too heterogeneous in design and outcome measures to perform a formal meta-analysis.

**Table 1.** Patient Demographics and Characteristics.

Author (Year)	Location	Journal	Study level	Study design	n	Number of shoulder arthroplasties	Sex, M:F	Age (years.)^a^	Side, L:R	Diagnosis (OA; ON; RA)	Parkinson's severity (I-V)^b^	Preop duration of symptoms (months)^a^	Shoulder replacement type	Glenoid (cemented)	Humerus (cemented)	Rotator cuff repair	Follow-up (years.)^a^	MINORS score
Koch et al^22^ (1997)	Mayo Clinic, USA	JSES	4	Retrospective Case Series	15^c^	16	6; 9	66 (49 -84)	7; 9	12; 3; 1	2 I; 6 II; 5 III; 1 IV; 1 V	44 (4 -108)	TSA (12 Neer; 4 Cofield Implants)	12 out of 16	3 out of 16	1 out of 16	5.3 (1.2-15 years)	11
Kryzak et al^23^ (2009)	Mayo Clinic, USA	JSES	3	Retrospective Cohort Study	Unknown	49	32; 17	69.7 (54 -87)	20; 29	Not described	NA	Not described	TSA (31 Cofield; 13 Neer; 4 Tournier; 1 Biomet	41 out of 49	4 out of 49	3 out of 49	8^d^ (years)	11
Kryzak et al^24^ (2010)	Mayo Clinic, USA	CORR	4	Retrospective Case Series	7	8	5; 2	72 (60-84)	5; 2	Fracture	1 II; 6 III	NA	Hemiarthroplasty (6 Neer; 1 Cofield)	NA	6 out of 7	NA	9.9 (years)	12
Dunn et al^27^ (2011)	Michigan; Brigham & Women's, USA	AmJOrth	4	Retrospective Case Series	3	3	0; 3	75	0; 3	2 Revision; 1 Fracture	NA	NA	Reverse Total Shoulder Arthroplasty	0 out of 3	1 out of 3	NA	17 months	5
Burrus et al^25^ (2015)	Virginia, USA	JSES	3	Retrospective Cohort Study	7032	7032 with PD (107,178 Total incl controls)	2874; 4158	445 < 65; 4586 65-79; 2000 >/= 80	Unknown	Unknown	Unknown	Unknown	Unknown	Unknown	Unknown	Unknown	Unknown	16
Cusick et al^26^ (2017)	Florida, USA	Orthopedics	4	Retrospective Case Series	10	10	4; 6	76 (63 -85)	Unknown	Unknown	Stage I and II only	Unknown	Reverse Total Shoulder Arthroplasty	Unknown	Unknown	Unknown	43 months (24-128)	18
Borbas et al^28^ (2021)	Zurich, Switzerland	Orthopedics	4	Retrospective Case Series	17	17	7; 10	73.9 (±9.5 years)	Unknown	Unknown	Unknown	Unknown	Reverse Total Shoulder Arthroplasty	Unknown	Unknown	Unknown	65 months	18
Moore et al^29^ (2022)	Weill Cornell; Yale, USA	JSES	3	Retrospective Case-Control	478	478 with PD (5193 Total incl controls)	2602; 2591	71 (±6 years)	Unknown	OA	Unknown	Unknown	TSA	Unknown	Unknown	Unknown	60 months	18

Abbreviations: CORR, clinical orthopaedics and related research; JSES, Journal of Shoulder and Elbow Surgery; TSA, total shoulder arthroplasty.

^a^ Values are reported as mean ± SD or mean (range).

^b^ Parkinson's Disease Severity = Hoehn and Yahr Classification of severity of Parkinson's Disease

^c^ 15 patients underwent 16 shoulder arthroplasties.

^d^ 43 patients.

**Table 2.** Clinical Outcomes.^a^

	Preoperative ROM				Postoperative ROM				Result (Unsatisfactory/Satisfactory/Excellent)	Positive association with findings	Survival-free revision at 10 years
Author (Year)	Pain	ABD	ER	IR	Pain	ABD	ER	IR			
Koch et al^22^ (1997)	10 moderate, 6 severe	106 (40-160, 32)	30 (0-80, 29)	NA	10 Slight, 5 moderate, 1 severe	96 (30-160, 36)	46 (15-85, 27)	NA	Unsatisfactory 10/16 (62.5%); 2/16 (13%); 4/16 (25%)	Age	
Kryzak et al^23^ (2009)	4.6 Pain Score	100	21	Sacrum	1.8 Pain Score (1-5)	119	44	Fifth Lumbar Vertebra	Unsatisfactory 20/49 (47%); 13/49 (30%); 10/49 (23%)	Age, gender, stage of PD	88% (95% CI: 78-99%)
Kryzak et al^24^ (2010)	NA	NA	NA	NA	2.5 Pain Score (1-5)	97	38	Sacrum	NA	NA	NA
Dunn et al^27^ (2011)	Unknown	NA	NA	NA	<1 (0-2) VAS Scale out of 10	n	13	NA	NA	NA	NA
Burrus et al^25^ (2015)	Unknown	Unknown	Unknown	Unknown	Unknown	Unknown	Unknown	Unknown	Unknown	NA	NA
Cusick et al^26^ (2017)	6.3 (VAS 1-10)	47.5	23.3	1.3	3.9	80	16	2.3			
Borbas et al^28^ (2021)											
Moore et al^29^ (2022)	Unknown	Unknown									97.2% with PD; 97.7% of controls at 5 years

Abbreviation: PD, Parkinson's disease.

^a^ Mean values used then range then SD.

**Table 3.** Complications and Reoperations.

Author (Year)	Type of shoulder replacement	Case group complication	Matched controls complication	Statistical comparison	Type of complication	Radiographic abnormality	Radiographic abnormality	Reoperation	Reoperation reason
				OR (95% CI)					
Koch et al^22^ (1997)	aTSA	16/16 (100%)			subluxation, loosening	16/16 (100%)	9 superior subluxation, 3 asymptomatic humeral loosening, 2 painful subluxations, 2 glenoid loosening	3/16 (19%)	2 painful subluxations, 1 glenoid loosening
Kryzak et al^23^ (2009)	aTSA	Unknown			loosening, subluxation	Unknown	Glenoid prosthetics loosening 30, glenoid shift 3 shoulders, 20 subluxation, 11 humeral periprosthetic radiolucency	8/49 (16%)	4 loosening components, 3 instability, 1 periprosthetic fracture
Kryzak et al^24^ (2010)	HA	Unknown			subluxation, non-union, mal-union	Unknown	4 glenohumeral subluxation, 1 greater tuberosity non-union; 3 superior malunion of greater tuberosity	0/7 (0%)	NA
Dunn et al^27^ (2011)	rTSA	3 out of 3 (100%)			scapular notching	3 out of 3 (100%)	3 scapular notching, Nerot Grade II in 2 cases and Grade III in 1 case (Nerot 0-4 Scale)	0/3 (0%)	NA
Burrus et al^25^ (2015)	Mixed				multiple, see below				
	aTSA	883/3390 (26%)	9012/47034 (19%)						
		55 (1.6)	524 (1.1)	1.5 (1.1-1.9)	Infection				
		136 (4)	768 (1.6)	2.5 (2.1-3.0)	Dislocation				
		190 (5.6)	1597 (3.4)	1.7 (1.4-2.0)	Revision				
		237 (7)	3506 (7.5)	0.9 (0.8-1.1)	Stiffness				
		34 (1)	317 (0.7)	1.5 (1.1-2.1)	Fracture				
		39 (1.2)	371 (0.8)	1.5 (1.1-2.0)	Loosening				
		192 (5.7)	1929 (4.1)	1.4 (1.2-1.6)	Systemic				
									
	rTSA	304/809 (38%)	3655/14262 (26%)						
		27 (3.3)	280 (2.0)	1.7 (1.2-2.6)	Infection				
		45 (5.6)	399 (2.8)	2.0 (1.5-2.8)	Dislocation				
		33 (4.1)	334 (2.3)	1.8 (1.2-2.6)	Revision				
		59 (7.3)	1051 (7.4)	1.0 (0.8-1.3)	Stiffness				
		11 (1.4)	140 (1.0)	1.4 (0.8-2.6)	Fracture				
		129 (15.9)	1451 (10.2)	1.7 (1.4-2.0)	Loosening				
					Systemic				
									
	HA	891/2833 (31%)	8915/38850 (23%)						
		66 (2.3)	593 (1.5)	1.5 (1.2-2.0)	Infection				
		145 (5.1)	736 (1.9)	2.8 (2.3-3.4)	Dislocation				
		158 (5.6)	1540 (4.0)	1.4 (1.2-1.7)	Revision				
		209 (7.4)	2968 (7.6)	1.0 (0.8-1.1)	Stiffness				
		91 (3.2)	861 (2.2)	1.5 (1.2-1.8)	Fracture				
		35 (1.2)	251 (0.6)	1.9 (1.3-2.7)	Loosening				
		187 (6.6)	1966 (5.1)	1.3 (1.1-1.5)	Systemic				
									
Cusick et al^26^ (2017)	rTSA								
	With Parkinson's disease	4/10 (40%)			fracture, baseplate failure, instability		2 acromial fracture, 1 glenoid baseplate failure, 1 post-operative instability	1/10 (10%)	Instability
	Without Parkinson's disease		6/40 (15%)					2/40 (5%)	Baseplate failure; dislocated polyethylene
									
Borbas et al^28^ (2021)	rTSA	35%	6%					29%	
									
Moore et al^29^ (2022)	aTSA	24.70% at 90 days	9.4% at 90 days		UTI, Pneumonia; Periprosthetic dislocation				

A total of 7613 shoulder arthroplasties in patients with Parkinson's disease were included in the systematic review. The majority of patients were aged between 65 and 79 years of age with a mean age of 72 in 7 of the 8 studies (Table 1). Three studies^22,23,29^ analyzed aTSA, 3 studies^26–28^ analyzed rTSA, 1 study analyzed HA, and the largest study had a mix of aTSA, rTSA, and HA patients (Table 3). Five studies^22–24,26,28^ objectively measured pain scores, and there was a reduction in pain scores postoperatively in 4^22,23,26,28^ of the studies which used an aTSA or a rTSA. In the HA paper,^24^ 4 of the 7 patients still had significant pain post-operatively. Range of motion was objectively measured in 4 studies,^22,23,26,35^ and mean scores improved in all studies except for abduction in one study^22^ (Table 2). Patient-reported outcome measures were utilized in 2 studies^22,23^ with 62.5% and 47% of patients unsatisfied with the outcome postoperatively.

Complication rates ranged from 26% to 100%. Two small studies^22,27^ reported complications in 100% of patients. The largest study^25^ with matched controls reported complications as per the type of shoulder replacement. aTSA patient's with Parkinson's disease had a 26% complication rate versus 19% with matched controls, rTSA patient's with Parkinson's disease had a 38% complication rate versus 26% with matched controls, and HA patient's with Parkinson's disease had a 31% complication rate versus 23% for matched controls. Complications included infection, dislocation, revision, stiffness, fractures, loosening, and systemic medical complications.

The most recent study^29^ included a large amount of patients (n = 478) with Parkinson's disease who underwent aTSA. It reported an implant survival at 5 years comparable between the case and control group at 97%. However, 90-day adverse events were significantly higher in Parkinson's patients with an overall complication rate of 24% compared to 9.4% in controls (n = 4715). The quality of evidence as measured by the average MINORS score was 11, which indicates a “fair” quality of evidence (Table 4).

**Table 4.** MINORS Criteria Summary.

Authors	MINORS 1: Clearly Stated Aim	MINORS 2: Inclusion of Consecutive Patients	MINORS 3: Prospective Collection of Data	MINORS 4: Endpoints Appropriate for Aim	MINORS 5: Unbiased Assessment of Endpoints	MINORS 6: Appropriate Follow-Up Period	MINORS 7: Loss to Follow Up	MINORS 8: Prospective Calculation of Study Size	MINORS 9: Adequate Control Group	MINORS 10: Contemporary Groups	MINORS 11: Baseline Equivalence Groups	MINORS 12: Adequate Statistical Analysis	MINORS Total Score
Koch et al^22^ (1997)	2	2	0	2	0	2	2	0	0	0	0	1	11
Kryzak et al^23^ (2009)	2	2	0	2	0	2	1	0	0	0	0	2	11
Kryzak et al^24^ (2010)	2	2	0	2	0	2	2	0	0	0	0	2	12
Dunn et al^27^ (2011)	2	0	0	1	0	1	1	0	0	0	0	0	5
Burrus et al^25^ (2015)	2	2	0	2	0	1	1	0	2	2	2	2	16
Cusick et al^26^ (2017)	2	2	0	2	0	2	2	0	2	2	2	2	18

## Discussion

Koch et al^22^ highlight the challenges posed by patient's with Parkinson's disease. They analyzed 16 arthroplasty surgeries between 1979 and 1990. Only 4 of the 16 patients received excellent results and most were unsatisfactory. Glenohumeral joint subluxation occurred in 9 of 16 (56%) arthroplasty surgeries. Most subluxations were asymptomatic but 2 required revision due to pain. Two additional patients had loosening of the glenoid component in association with superior subluxation, with 1 requiring revision surgery. Therefore, 3 of 16 (19%) patients underwent revision surgery. Most of the patients in this series had intact rotator cuffs.

The increase in subluxations was likely a result of increased tone of the shoulder girdle musculature, the difficulties with rehabilitation, and stretching of the rotator cuff-capsule arthrotomy site, particularly the rotator interval. The uncertainty in this patient cohort was highlighted in 1 patient who underwent bilateral shoulder arthroplasties in whom the extremes of results were achieved. It is highlighted that relief of pain is reliable in patients with Parkinson's disease who undergo shoulder arthroplasty but poor functional results are analogous to patients with rheumatoid arthritis who undergo shoulder arthroplasty.

The overall design of aTSA has evolved since before the 1990s when Koch et al^22^ published their paper. Although it must be noted that joint subluxation was present in 9 of 16 (56%) of their patients, only 2 of these patients ultimately required revision surgery due to subluxation. Technology and techniques have evolved and outcomes have improved regarding aTSA. Although the risk of subluxation is present with an aTSA in Parkinson's disease, aTSA is an unconstrained implant where subluxation is inherent to its intended design principles.

Kryzak et al^23^ is a follow-on study from the same institution as Koch et al.^22^ This study examined shoulder arthroplasties performed between 1978 and 2005 at the same institution, thus including 15 additional years of shoulder arthroplasty patients with Parkinson's disease. There were a total of 8/49 (16%) patients who underwent revision shoulder arthroplasty, which is the same percentage as the original study. The authors note that 46.5% (20/43) of shoulders that could be evaluated radiographically demonstrated glenohumeral subluxation. Moderate–severe subluxation was found to have a significantly higher level of pain compared to patients with none or mild subluxation. Postoperative abduction was lower in patients with moderate to severe postoperative subluxation. Glenoid periprosthetic radiolucency was present in 69.8% (30/43) of shoulders, while humeral periprosthetic radiolucency was present in 25.6% (11/43) of shoulders.

The results of Koch's study^22^ demonstrate that total shoulder arthroplasty can provide good pain relief but with poor functional results. Similar findings were noted for hip and knee surgery in patients with Parkinson's disease.^16–34^ Kryzak et al^23^ highlight the importance of minimizing associated complications by having Parkinson's disease as well controlled as possible prior to surgery, with a recent neurology evaluation. The disease should be mild overall with minimal rigidity about the shoulder. The social situation should allow for adequate care postoperatively.

The authors also note the importance of ruling out a concurrent rotator cuff tear or any signs of dementia. Kryzak et al^23^ suggest if there is moderate or severe subluxation preoperatively, it is unlikely that shoulder arthroplasty will be able to control it. They concluded that total shoulder arthroplasty is associated with significant long-term improvement in pain, external rotation, and abduction in patients with Parkinson's disease, however, early postoperative instability is higher in this population.

Kryzak et al^24^ reviewed 7 patients with Parkinson's disease who underwent shoulder HA due to a fracture of the proximal humerus. No patient underwent revision surgery. Mean postoperative pain scores were 2.5 (1-5 scale). However, the benefit of HA was marginal with 3/7 shoulders having persistent pain and limited function postoperatively.

Dunn et al^27^ published the first case series of rTSA in 3 patients with Parkinson's disease. This was a heterogeneous group of patients with Parkinson's disease. The authors reached a similar conclusion in stating falls, weak musculoskeletal structures, and onset of dementia further limit functional benefits in these patients. Pain was reduced in the series but functional recovery was poor. Scapular notching was noted in each of the 3 cases and although not possible to prove the causality of Parkinson's disease and notching, the authors state it is conceivable notching was secondary to the Parkinson's disease characteristics of rigidity, tremor, bradykinesia, and akinesia.

Burrus et al^25^ performed a retrospective cohort study with a significantly larger sample size than previous studies. The authors matched Parkinson's disease patients undergoing aTSA, rTSA, and HA to controls. They concluded that Parkinson's disease is associated with increased rates of infection, dislocation, revision shoulder arthroplasty, fracture, component loosening, and systemic complications after conventional aTSA, rTSA, and HA (Table 3). The MediCare database was searched from 2005 to 2012 for shoulder arthroplasty cases in patients with Parkinson's and without Parkinson's disease. Age, gender, obesity, diabetes, and tobacco use were used to match groups.

Burrus et al^25^ state that patients with Parkinson's disease have higher complications secondary to reasons already mentioned such as osteoporotic bone, increased falls risk, rigidity and tremor, abnormal soft tissues, and asynchronous motor function. There is an increased risk of complications such as urinary tract infections, pneumonia, confusion, and decubitus ulcers and a 6-month mortality of up to 47% for patients with Parkinson's disease for patients undergoing total hip or knee and hip fracture surgery.^35^ A significant finding in Burrus et al^25^ is that no difference between the cohorts when controlling for obesity, diabetes, and tobacco use. These 3 medical comorbidities have been clearly associated with complications in shoulder arthroplasty. This accentuates the importance of the disease process with Parkinson's disease in shoulder arthroplasty patients and the increase in complications.

Burrus et al^25^ is the first study that has shown an increased risk of periprosthetic joint infection in patients with Parkinson's disease undergoing shoulder arthroplasty surgery. This may be due to the fact that Parkinson's disease patients who are hospitalized have a higher risk of urinary tract infection and pneumonia compared to the general population.^36^ Wound complications in Parkinson's disease patients is also noted to be higher than the general population.^36^

Cusick et al^26^ have identified there is a lack of evidence for the choice of shoulder arthroplasty in Parkinson's disease patients. The authors suggest that early instability and unsatisfactory results in unconstrained shoulder arthroplasty appear to be higher for Parkinson's disease patients and a rRSA, which provides inherent constraint, maybe a more appropriate treatment option.^26^ The authors argue the rTSA has provided safe and effective results for patients with a variety of shoulder pathologic conditions in which there is an unstable fulcrum of the glenohumeral joint.^26^

Ten patients with Parkinson's disease who underwent rTSA between 2004 and 2011 were identified. These patients were matched to a cohort of 40 patients without Parkinson disease. The Hoehn and Yahr Classification system was used, and only grades I and II patients were included in the study. Patients were matched with controls regarding their preoperative diagnosis including rotator cuff arthropathy, osteoarthritis, failed HA, and failed total shoulder arthroplasty.

Patients with Parkinson disease had statistically significant improvements in postoperative ASES (American Shoulder and Elbow Score) scores and approaching significance in VAS and ASES pain scores. They did not show a statistical improvement in ASES function scores. Control patients had improvements in all scores. Parkinson's disease patients only demonstrated statistically significant improvement in forward elevation, whereas control patients showed improvements in all planes of motion. Complications occurred in 4 of 10 (40%) of patients with Parkinson's disease, whereas patients without Parkinson's disease had 6 of 40 (15%) complications. Three of the 4 patients with Parkinson's disease were treated nonoperatively. One patient who had deltoid spasticity was treated with botulinum toxin type A injection; another patient was revised due to a first instability episode and was managed conservatively after a second instability episode. In the control group, there were 4 acromial fractures that were treated nonoperatively, one failed baseplate due to a broken central screw, and one case of instability with an associated dislocated polyethylene insert, which was both revised.

Cusick et al^26^ recommended a multidisciplinary approach to shoulder arthroplasty in Parkinson's disease patients. Those with more advanced diseases should not be considered candidates for surgery, preoperative optimization by the neurologist, and close follow-up postoperatively for optimal control of symptoms, which can translate into improved outcomes. The role of intraoperative or postoperative botulinum toxin type A injections is still unclear.

Borbas et al^28^ demonstrated that outcome scores do improve for Parkinson's disease patients after a rTSA. However, the range of motion is worse in this cohort compared to matched controls without Parkinson's disease. Postoperative complications of 35% versus 6% and reoperation rate of 29% versus 1% were substantially greater for the patients with Parkinson's disease.

Moore et al^29^ showed that patient's undergoing an aTSA had a 3-fold higher odds of periprosthetic dislocation in the 90-day postoperative period but equivalent rates of other short-term adverse events as well as implant survival at 5 years.

## Conclusion

Patients with Parkinson's disease undergoing shoulder arthroplasty achieved similar pain relief but inferior clinical function to those without Parkinson's disease. Range of motion was less predictable, and complication rates were significantly higher in Parkinson's disease patients. This study provides the surgeon and patient with useful data regarding the expected clinical outcomes and potential complications of shoulder arthroplasty in this patient cohort, which should assist in the shared decision-making process regarding shoulder replacement surgery.
